# Onychomycosis Caused by* Fusarium* spp. in Dakar, Senegal: Epidemiological, Clinical, and Mycological Study

**DOI:** 10.1155/2017/1268130

**Published:** 2017-12-04

**Authors:** Khadim Diongue, Mouhamadou Ndiaye, Mame Cheikh Seck, Mamadou Alpha Diallo, Aïda Sadikh Badiane, Daouda Ndiaye

**Affiliations:** ^1^Laboratoire de Parasitologie-Mycology, CHU Le Dantec, BP 5005, Dakar, Senegal; ^2^Service de Parasitologie-Mycology, Faculté de Médecine, de Pharmacie et d'Odontologie, Université Cheikh Anta Diop, BP 16477, Dakar, Senegal

## Abstract

*Fusarium* spp. represent 9 to 44% of onychomycoses caused by* fungi* other than dermatophytes. This retrospective study describes 17 cases of* Fusarium* onychomycosis diagnosed at the Laboratory of Parasitology and Mycology of Le Dantec University Hospital in Dakar, Senegal, from 2014 to 2016. It included all patients received in the laboratory for suspicion of onychomycosis between January 1, 2014, and December 31, 2016. Diagnosis was based on mycological examination including direct examination and culture. Mycological analysis was considered positive when direct examination and culture were positive after at least one repeat. Seventeen* Fusarium* onychomycosis cases representing 12.9% of all onychomycoses reported were diagnosed. There were 5 cases on the fingernails and 12 on the toenails in 6 males and 11 females, and the mean age was 44 years (range: 26–64). Onychomycoses were diagnosed in immunocompetent patients except in a diabetic patient. The mean duration of lesions was 4.9 years (range: 1–15), and distal subungual onychomycosis was predominant. Almost all patients were from suburban areas of Dakar region. The most frequent species isolated belong to* Fusarium solani* complex. Because of the risk of disseminated infection in immunocompromised patients, realization of susceptibility tests is necessary to ensure better therapeutic management.

## 1. Introduction

The genus* Fusarium*, described for the first time in 1809, contains saprophyte telluric species and plant pathogens. These organisms are also involved in human pathology, causing mycotoxicoses and infections which can be locally invasive or disseminated. Very cosmopolitan,* Fusarium* is found in tropical areas, temperate regions, deserts, and mountainous and even arctic zones [1].

Currently, the genus* Fusarium* comprises at least 300 phylogenetically distinct species, 20 species complexes, and nine monotypic lineages. Most of the identified opportunistic* Fusarium* pathogens belong to the* F. solani* complex (FSC),* F. oxysporum* complex (FOC), and* F. fujikuroi* complex (FFC) [2]. Among immunocompetent patients, tissue breakdown (as caused by trauma, severe burns, or foreign bodies) is the risk factor for fusariosis. Infections include keratitis, onychomycosis, and occasionally peritonitis and cellulitis [3].

Frequently, walking barefoot is the main cause for* Fusarium* onychomycoses, and they preferentially infect the big toe. They may be superficial or subungual. According to studies, they represent 9 to 44% of onychomycoses caused by* fungi* other than dermatophytes. Although* Fusarium* onychomycosis remains mostly localized, it could also represent the portal of entry for disseminated diseases in immunocompromised patients [1]. The genus* Fusarium* is ubiquitous in the environment and can hang on to the nail plate especially in case of dystrophy or local trauma. This mold may be just saprophytic or truly pathogenic, provided it is found repeatedly on multiple samples [4].

Here, we describe 17 cases of* Fusarium* onychomycosis diagnosed in the Laboratory of Parasitology and Mycology of Le Dantec University Hospital in Dakar, Senegal, from 2014 to 2016.

## 2. Patients and Methods

We conducted a retrospective and descriptive study including all patients received in the laboratory for suspicion of onychomycosis between January 1, 2014, and December 31, 2016.

Diagnosis was based on mycological examination including direct examination and culture as described in a previous article [5]. A microscopic direct examination of all specimens was carried out in 20% KOH solution. The specimens were cultured in 2 plates/tubes, one containing Sabouraud-chloramphenicol dextrose agar and the other containing Sabouraud-chloramphenicol cycloheximide. Cultures were incubated at 22–27°C and evaluated for growth after 48 h and then once weekly for a month. The specimen was considered positive when microscopic examination and culture were positive. On the other hand, when* Fusarium* sp. was isolated alone, to assert its pathogenicity, rigorous criteria were used. They include the following [6]:

- Positive direct (or histological) examination is carried out.
- Culture (preferably in Petri dishes rather than in tubes, so as not to miss out on an association with a dermatophyte) must show the growth of the fungus at the level of (almost) all the seeding points (and not elsewhere in the agar).
- It is strongly recommended to renew the samples collection at the same sites, in order to verify the isolation of the same fungus.

Identification of* fungi* was based on the speed of growth and especially on the macroscopic and microscopic characteristics of the colonies and sometimes on their physiological (germ tube test) and biochemical (urease test) characteristics [2, 7–10].

Data were recorded in Microsoft® Excel 2007 and transferred into Epi Info® 7 where statistical analysis was done.

## 3. Results

**Figure 1:**
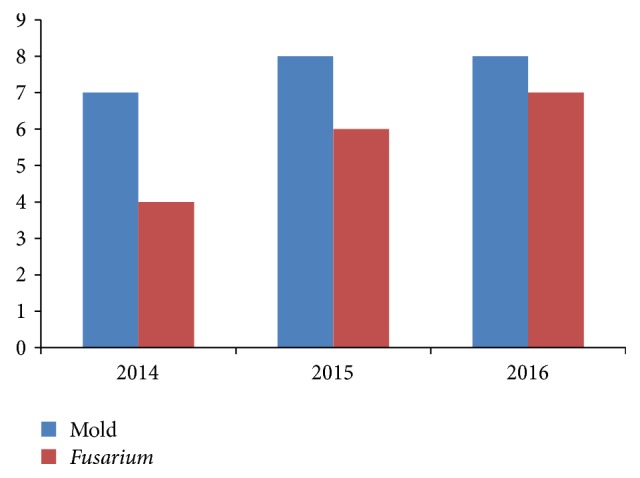
Evolution of the cases of mold and* Fusarium* onychomycoses throughout years.

During the study period, 132 cases of onychomycosis were diagnosed.  Figure 1 shows the case number evolution of both the mold and the* Fusarium *genus. Cases caused by* Fusarium* species were 17 (12.9%) in 6 males and 11 females, and the mean age was 44 years (range: 26–64).

Table 1 shows the clinical and demographic findings and mycological details of these 17 cases. The most frequent isolated species belong to the FSC with 8 cases. Two concomitant infections were observed where* Fusarium* was associated with* Candida albicans *(cases 15 and 16).

Onychomycoses were diagnosed in immunocompetent patients except in a diabetic patient (case 6). Almost all patients were from Dakar region and 2 cases were from Saint Louis and Thiès regions.

The lesions were onychomycosis alone in 9 cases, while in 7 cases, onychomycosis was associated (due to the same* Fusarium*) with interdigital* tinea pedis* and in one case with interdigital and chronic hyperkeratotic (moccasin)* tinea pedis* (case 13).

The mean duration of lesions was 4.9 years (range: 1–15). Concerning the location of onychomycoses, 5 were localized at the fingernails whereas 12 were at the toenails and 3 among the latter were on the big toe (cases 1, 8, and 17). Regarding the type of attack, onychomycosis was distal subungual in 7 cases, onycholysis in 2 cases, proximal with paronychia in 1 case, and secondary to interdigital* tinea pedis* in 7 cases.

The majority of cases were treated with terbinafine (tablet and/or cream) according to what we have reported.

## 4. Discussion

*Fusarium* onychomycoses are not rare since* Fusarium* spp. were reported to be the causative agent of 9–44% of nail invasions caused by nondermatophytic molds [4]. Several authors have shown the presence of* Fusarium* spp. as agents of onychomycosis, with a frequency in the range of 0.97–6% [11]. Our prevalence of 12.9% is outside this range, but it is very similar to that reported in a study conducted in Lyon (France) between 2008 and 2010, showing a prevalence of 12.6% [12]. In contrast, in other studies carried out around the world,* Fusarium* onychomycosis was diagnosed with prevalences within this range, with 0.09% in Tunis (Tunisia) between 1996 and 2010 [13], 3.1% in Guatemala (Guatemala) between 2008 and 2011 [14], 6.25% in Galle (Sri Lanka) published in 2008 [15], and 7.3% in Kanpur (India) between June and October 2011 [16].

*Fusarium* onychomycoses were exclusively diagnosed in adults with a mean age of 44 years (range: 26–64), and there was a predominance of females. The first observation has already been reported in a report of seven cases from Natal (Brazil) between 2002 and 2004 with an average age of 47 years (range: 31–66) [17]. Likewise, Ranawaka et al. found* Fusarium* onychomycoses in patients with ages ranging between 18 and 74 years with a mean of 43 years [15]. The high rate of isolation in females may be the result of the continuous use of open shoes, with a higher risk of injuries and contact with soil [11]. According to Chabasse and Pihet, frequencies of* Fusarium* onychomycoses increase with age. Thus, the frequency is between 15 and 20% in adults over 40 and exceeds 30% in people over 70 years of age. This prevalence, clearly superior in the elderly, is related to the following factors: smaller nail growth, bad blood circulation in the lower limbs, physiological immunosuppression related to age, ungual microtrauma, and sometimes inability to provide adequate feet care [6].

The majority of cases (12/17) were located in toenails. This toenail predominance of* Fusarium* onychomycosis was reported in Brazil [17] and also in Sri Lanka, where a particular predominance was also noted in the big toenail [15]. We found this location on the big toenail in three cases. These observations could be explained because contamination occurs from soil, especially in individuals who walk with open sandals or barefooted [3]. This practice is all the more a risk factor in Senegal, where, apart from the city centers of the regions, all the streets are sanded. This would justify the fact that 15 of the 17 patients come from suburban areas of Dakar, other than the absence of a mycology laboratory in these regions.

With the increasing number of immunocompromised patients, many species of* fungi* originally regarded as laboratory contaminants are now considered to be agents of mycosis and may sometimes also affect immunocompetent patients [11]. This is the case of* Fusarium* species. In our series, no immunodeficiency was noted in 16 of the 17 patients. Only one case of diabetes was observed, which probably contributed to the presence of microconidia at the direct examination. It is a rare observation but one we have already observed with* Fusarium* sp. The first case of interdigital* tinea pedis* due to* Fusarium* that we reported in Dakar had also shown this with hyphae associated with microconidia [18]. Néji et al. also showed a direct examination of* Fusarium* onychomycosis showing hyphae associated with sickle-shaped macroconidia [13].

The most common clinical presentations were distal subungual onychomycosis and onycholysis. This is contrary to what has been published by Dignani and Anaissie, who stipulated that the most common clinical presentations include proximal subungual onychomycosis with or without paronychia [3]. However, distal subungual attack was observed by Calado et al., in 6/7 cases [17]. These authors found the proximal subungual lesion associated with paronychia in one case like our results. We observed onychomycosis secondary to interdigital* tinea pedis* with the same proportion as the distal subungual attack. This association is frequent and could be explained by the anatomical proximity and mostly because interdigital* tinea pedis* is a rare motive of consultation in Dakar [5].

We found a mean duration of lesions of 4.9 years (range: 1–15). A similar history of infection has been reported in the report of seven cases of* Fusarium* onychomycosis from Natal (Brazil) showing a mean of 5 years, range from 8 months to 10 years.

According to the literature, the most common* Fusarium* species responsive to human infections are* F. solani* (FSC, 50%),* F. oxysporum* (FOC, 14%),* F. verticillioides* (FFC, 11%), and* F. moniliforme* (FFC, 10%) [1]. The species found in our series are roughly the same mentioned above with the same order of medical importance except for* F. lichenicola* (FSC) which was isolated with the same proportion as* F. solani* and* F. oxysporum*.* Fusarium oxysporum* is the species most often isolated in toenails, while* F. solani* predominates in fingernails [6]. This repartition of the two species was noted mainly but not always. In contrast, in other studies carried out in Brazil, Calado et al. found only* F. solani* on 6 toenails and 1 fingernail onychomycosis [17]. Likewise, another study in Brazil showed that, out of ten cases of* F. solani* onychomycosis, eight were located on toenails and only two were on fingernails [11]. Among other species, we found* F. moniliforme* (FFC) with two cases like in the study of Ranawaka et al. in Sri Lanka [15] and* F. subglutinans* (FFC) in one case where other authors have found other species [11].

Concerning the treatment by terbinafine, it is explained that this molecule is among the rare available antifungals in Senegal, in a tablet form, with fluconazole. However, it is demonstrated that fluconazole is weakly active or inactive on filamentous* fungi* such as* Aspergillus* and* Fusarium *[19, 20].

## 5. Conclusion

The prevalence of* Fusarium* onychomycoses diagnosed in Dakar in the period 2014–2016 was not low and we remark that the number of cases increases throughout years. These infections were predominant in adults, especially in females. They are mostly located in toenails with a nonnegligible association with interdigital* tinea pedis* and affect immunocompetent patients. Although* Fusarium* onychomycosis is usually localized in immunocompetent individuals, it could also represent the portal of entry for disseminated diseases in immunocompromised patients such as diabetics. Hence, there is a need to carry out susceptibility tests to ensure better therapeutic management.

## Figures and Tables

**Table 1:** Clinical and demographic findings and mycological details of infection in 17 cases of onychomycosis caused by *Fusarium* species.

Number	Sex/age (years)	Location	Region of origin	Attack type	Duration (years)	Immune status	DE	Species
*(1)*	*F/43*	Big toenail	*Dakar*	Distal subungual	15	*IC*	*Hyp*	FSC
*(2)*	*F/49*	Fingernails	*Dakar*	Onycholysis	2	*IC*	*Hyp*	FSC
*(3)*	*F/47*	Toenails	*Dakar*	Onycholysis	1	*IC*	*Hyp*	FOC
*(4)*	*M/36*	Fingernails	*Dakar*	Distal subungual	7	*IC*	*Hyp*	FSC
*(5)*	*F/56*	Toenails	*Dakar*	Secondary to ITP	NS	*IC*	*Hyp*	FOC
*(6)*	*F/64*	Toenails	*Dakar*	Distal subungual	8	*Diabetic*	*Mic*	FOC
*(7)*	*F/30*	Fingernails	*Dakar*	Distal subungual	3	*IC*	*Hyp*	FFC
*(8)*	*M/43*	Big toenail	*Dakar*	Distal subungual	7	*IC*	*Hyp*	FFC
*(9)*	*F/33*	Toenails	*Dakar*	Secondary to ITP	1,5	*IC*	*Hyp*	FSC
*(10)*	*M/55*	Toenails	*St. Louis*	Secondary to ITP	2	*IC*	*Hyp*	FSC
*(11)*	*M/36*	Fingernails	*Dakar*	Proximal paronychia	9	*IC*	*Hyp*	FFC
*(12)*	*F/39*	Toenails	*Dakar*	Secondary to ITP	1,8	*IC*	*Hyp*	FSC
*(13)*	*M/59*	Toenails	*Dakar*	Secondary to ITP	5	*IC*	*Hyp*	FSC
*(14)*	*F/44*	Fingernails	*Thiès*	Distal subungual	10	*IC*	*Hyp*	FOC
*(15)*	*M/63*	Toenails	*Dakar*	Secondary to ITP	5	*IC*	*Hyp*	FSC + *C. albicans*
*(16)*	*F/26*	Toenails	*Dakar*	Secondary to ITP	2	*IC*	*Hyp*	FFC + *C. albicans*
*(17)*	*F/26*	Big toenail	*Dakar*	Distal subungual	2	*IC*	*Hyp*	FFC

ITP: interdigital *tinea pedis*; NS: not specified; IC: immunocompetent; DE: direct examination; Hyp: hyphae; *Mic*: microconidia; *FSC*:* Fusarium solani* complex; FOC: *Fusarium oxysporum* complex; FFC: *Fusarium  fujikuroi* complex.
